# Screening of copy number variants in the 22q11.2 region of congenital heart disease patients from the São Miguel Island, Azores, revealed the second patient with a triplication

**DOI:** 10.1186/s12863-014-0115-6

**Published:** 2014-11-07

**Authors:** Renato Pires, Luís M Pires, Sara O Vaz, Paula Maciel, Rui Anjos, Raquel Moniz, Claudia C Branco, Rita Cabral, Isabel M Carreira, Luisa Mota-Vieira

**Affiliations:** Molecular Genetics and Pathology Unit, Hospital of Divino Espírito Santo of Ponta Delgada, EPE, Av. D. Manuel I, Ponta Delgada 9500-370, São Miguel Island, Azores Portugal; Centre for Biodiversity, Functional and Integrative Genomics (BioFIG), Faculty of Sciences, University of Lisboa, Lisboa, 1749-016 Portugal; Cytogenetics and Genomics Laboratory, Faculty of Medicine, University of Coimbra, Coimbra, 3000-354 Portugal; Department of Paediatrics, Hospital of Divino Espírito Santo of Ponta Delgada, EPE, Av. D. Manuel I, Ponta Delgada 9500-370, São Miguel Island, Azores Portugal; Department of Paediatric Cardiology, Hospital of Santa Cruz, Av. Prof. Dr. Reinaldo dos Santos, Carnaxide, 2790-134 Portugal; Instituto Gulbenkian de Ciência, Rua da Quinta Grande, 6, Oeiras, 2780-156 Portugal

**Keywords:** Congenital heart disease, 22q11.2 deletion, 22q11.2 microduplication, 22q11.2 triplication

## Abstract

**Background:**

The rearrangements in the 22q11.2 chromosomal region, responsible for the 22q11.2 deletion and microduplication syndromes, are frequently associated with congenital heart disease (CHD). The present work aimed to identify the genetic basis of CHD in 87 patients from the São Miguel Island, Azores, through the detection of copy number variants (CNVs) in the 22q11.2 region. These structural variants were searched using multiplex ligation-dependent probe amplification (MLPA). In patients with CNVs, we additionally performed fluorescent *in situ* hybridization (FISH) for the assessment of the exact number of 22q11.2 copies among each chromosome, and array comparative genomic hybridization (array-CGH) for the determination of the exact length of CNVs.

**Results:**

We found that four patients (4.6%; A to D) carried CNVs. Patients A and D, both affected with a ventricular septal defect, carried a *de novo* 2.5 Mb deletion of the 22q11.2 region, which was probably originated by inter-chromosomal (inter-chromatid) non-allelic homologous recombination (NAHR) events in the regions containing low-copy repeats (LCRs). Patient C, with an atrial septal defect, carried a *de novo* 2.5 Mb duplication of 22q11.2 region, which could have been probably generated during gametogenesis by NAHR or by unequal crossing-over; additionally, this patient presented a benign 288 Kb duplication, which included the *TOP3B* gene inherited from her healthy mother. Finally, patient B showed a 3 Mb triplication associated with dysmorphic facial features, cognitive deficit and heart defects, a clinical feature not reported in the only case described so far in the literature. The evaluation of patient B’s parents revealed a 2.5 Mb duplication in her father, suggesting a paternal inheritance with an extra copy.

**Conclusions:**

This report allowed the identification of rare deletion and microduplication syndromes in Azorean CHD patients. Moreover, we report the second patient with a 22q11.2 triplication, and we suggest that patients with triplications of chromosome 22q11.2, although they share some characteristic features with the deletion and microduplication syndromes, present a more severe phenotype probably due to the major dosage of implicated genes.

## Background

Congenital heart disease (CHD) affects about 12 in 1000 live births and includes structural heart defects which impair the cardiac function [1]. The pathogenesis of these defects is largely unknown, but it is widely reported that genetic factors play an important role in the complex aetiology of CHD [2]. Although much of these diseases are thought to have a complex genetic and/or environmental aetiology, an increasing number of families with monogenic CHD have been reported and disease-causing genes are being identified [3]. Additionally, recurrent copy number variants (CNVs) have been also found in a significant proportion of patients with CHD [2]. These structural variants are strongly associated with genetic syndromes [1]. The most common example is the 22q11.2 deletion syndrome, which is estimated to affect approximately 1 in 4000 live births [4] and results most commonly from a 3 Mb deletion in the 22q11.2 region [4-7]. This region contains approximately 40 genes [8], being the *TBX1* and *COMT* the most relevant [9]. Genomic rearrangements in the 22q11.2 region are due to the presence of several copies of low-copy repeat sequences (LCR22) that mediate inter-chromosomal (inter-chromatid) non-allelic homologous recombination (NAHR) [4,7,10].

The 22q11 deletion phenotype is highly variable and includes the following characteristics: cardiovascular anomalies; palatal abnormalities; nasal voice; immune deficiency related with hypoplasia of the thymus; endocrine dysfunctions, such as hypocalcaemia; a varying degree of cognitive defects and intellectual disability; velopharyngeal insufficiency; and a characteristic craniofacial dysmorphism [5,6,11,12].

Microduplications of 22q11.2 have recently been characterized as a new genomic duplication syndrome [8,13], by sharing some clinical features with the 22q11.2 deletion syndrome. They show an extremely variable phenotype, ranging from normal or mild learning disability to multiple congenital defects, such as: heart defects, velopharyngeal insufficiency with and without cleft palate, growth delay, mild dysmorphic features, and behavioural abnormalities [5,8,13-15]. The large majority of affected individuals have 3 Mb duplications of the typically deleted region including the *TBX1* gene [8]. Regarding other CNVs, Yobb and colleagues [14] reported one patient with a triplication of the 3 Mb 22q11.2 deletion syndrome region, and suggested that this structural variant only causes a mild phenotype.

In 2006, our research group [16] conducted the first population-based epidemiological study on congenital heart malformations in the São Miguel Island, where the results pointed out the great interest for genetic studies in order to understand the aetiology of these complex pathologies. Since 22q11.2 CNVs are frequently implicated in CHD, in the present work we assessed the presence or absence of these structural abnormalities in 87 patients, and explored the multiple clinical features of patients with CNVs. This study is the first molecular-based investigation of CHD in patients from the São Miguel Island.

## Results

### CHD patients with CNVs in the 22q11.2 region

In the São Miguel Island, an epidemiological study carried out by our research group [16] demonstrated that the prevalence of CHD was relatively high, reaching 9.16/1000 live births. However, understanding the genetic features underlying CHD in patients from this island remains unknown. In order to investigate the presence of CNVs in the 22q11.2 region of 87 CHD patients, we first performed a genetic screening using multiplex ligation-dependent probe amplification (MLPA) assay and, in the positive cases, we additionally tested by fluorescence *in situ* hybridization (FISH) and array comparative genomic hybridization (array-CGH). We detected CNVs in four patients (4.6%), one male (A) and three females (B, C, D).

Patient A is a 17 year-old male who has a ventricular septal defect (VSD). During the first month of pregnancy, and before knowing her pregnancy, his mother took medication for hypertiroidism. At eight months of age, the patient had febrile seizures, which remained after heart surgery. During childhood, he was diagnosed with developmental delay, hyperactivity, epilepsy, scoliosis, feet bunions, and urogenital abnormality. Patient B is a 20 year-old female who presents restrictive interventricular communication, membranous sub-aortic stenosis, cognitive deficit and facial dysmorphism. The patient’s family is endogamous, since her grandparents are from the same village. Her mother had an advanced age at conception (42 year-old) and her father presents mild intellectual disability with learning difficulties. Patient C is a 24 year-old female who has an atrial septal defect (ASD), facial dysmorphism, mild developmental delay, and learning difficulties. This patient is from a consanguineous family, since two of her great-great-grandparents were siblings. All of her four siblings had CHD and three of them died. Patient D is an 11 year-old female who has a VSD detected immediately after birth. She also has facial dysmorphism, developmental delay, moderate psychomotor impairment, and hypocalcaemia. At 15 days of age, this patient underwent cardiac surgery.

### MLPA analysis

The results showed that the 22q11.2 region was normal in 83 (95.4%) and abnormal in four patients (A to D; Table 1). In the patients with an abnormal region, we performed the analysis of the segments with structural variation. We detected a deletion of 14 probes, from the *CLTCL1* to the *LZTR1* genes (data not shown) in DNA samples of patients A and D. In order to determine if these deletions were inherited or acquired *de novo*, we further assessed the parents’ samples. This analysis revealed that the same region was normal in the parents of patients A and D, *i.e.*, without any CNV. In patient B, we found that the same 14 probes were in higher doses than duplication, suggesting the presence of either a double disomy (2:2, two chromosomes with 22q11.2 duplication) or a tetrasomy (1:3, one normal chromosome and a 22q11.2 triplication in the other). Among the patient’s parents, the 22q11.2 region was normal in her mother (1:1) and duplicated (1:2) in her father. Regarding patient C, the results evidenced a duplication of the 14 probes and of the *TOP3B* probe, while in her mother only the *TOP3B* was duplicated; her father was normal.

**Table 1:** **Summary of the screening of the 22q11.2 region in CHD patients and their parents**

**Patient**	**Person tested**	**Age (yrs)**	**22q11.2 region**	**Methods used**
A	Patient	17	Deletion (*de novo*)	MLPA, array-CGH
	Mother	47	Normal	MLPA
	Father	49	Normal	MLPA
B	Patient	20	Triplication (paternally inherited with extra copy)	MLPA, FISH, array-CGH
	Mother	61	Normal	MLPA, FISH
	Father	63	Duplication	MLPA, FISH, array-CGH
C	Patient	24	Duplication (*de novo*) + *TOP3B* duplication	MLPA, array-CGH
	Mother	55	Normal *+ TOP3B* duplication	MLPA
	Father	58	Normal	MLPA
D	Patient	11	Deletion (*de novo*)	MLPA, FISH, array-CGH
	Mother	38	Normal	MLPA
	Father	39	normal	MLPA

### Analysis of CNVs by FISH and array-CGH

**Figure 1:**
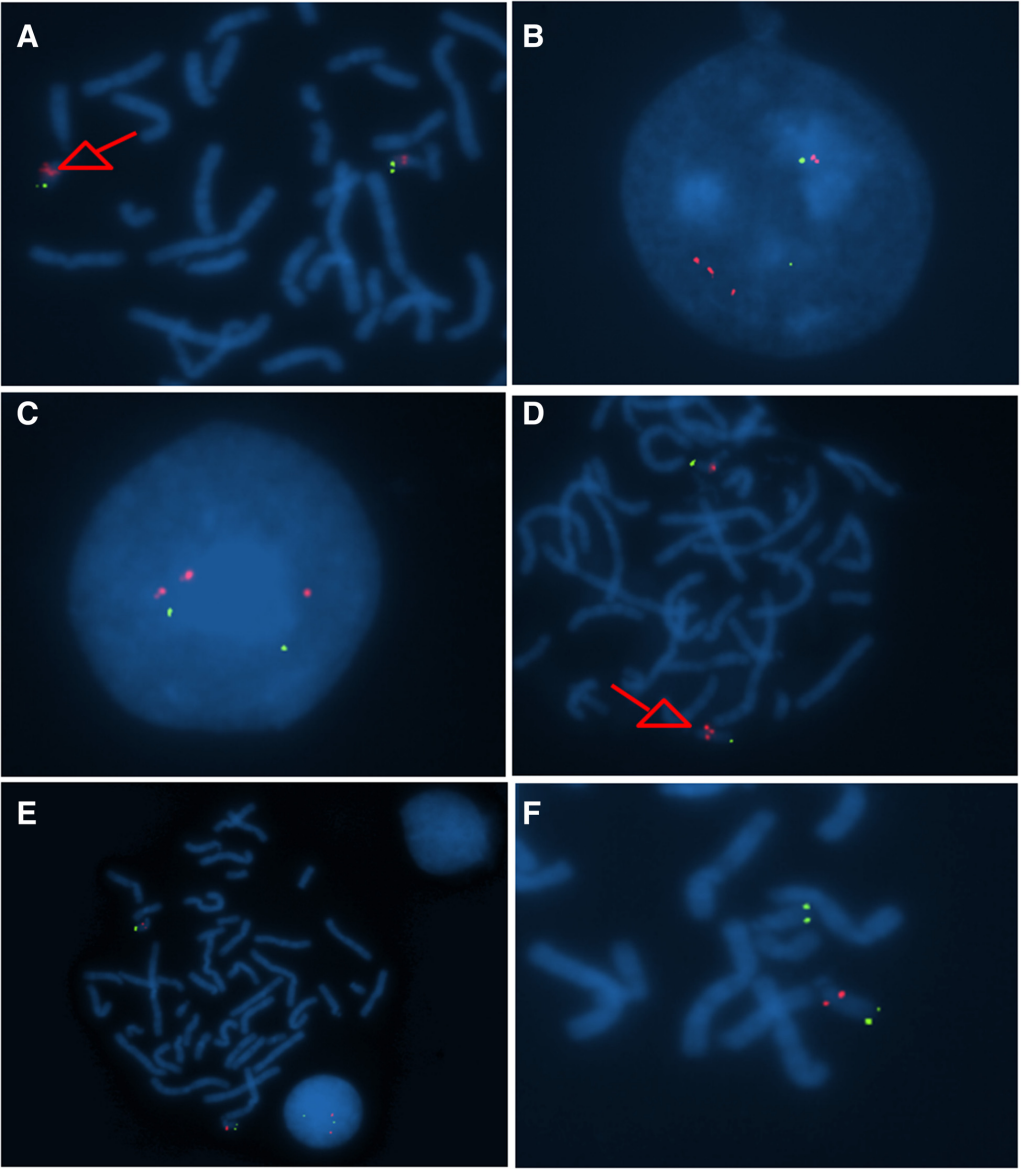
**Photographs of FISH assays.** FISH with specific red probe (N25) in patient B on the metaphase **(A)** and interphase **(B)** chromosomes. FISH image of the father of patient B on interphase **(C)** and metaphase **(D)**. **(E)** Interphase and metaphase FISH image with red probe in the mother of patient B. **(F)** Metaphase FISH image revealed in patient D.

We assessed the exact number of 22q11.2 copies among each chromosome by a molecular cytogenetic technique, namely FISH. The FISH results confirmed those obtained by MLPA in patients B and D. In patient B, FISH revealed a tetrasomy of 22q11.2 region, with one of the two chromosomes having a triplicated sign of probe N25 in metaphase (Figure 1A) and interphase (Figure 1B). The evaluation of this patient’s parents showed that the father carried a 22q11.2 duplication (Figures 1C and 1D), whose expansion could be the possible cause of the presence of triplication in his daughter (Figure 1B). A normal pattern was revealed in her mother’s homologous chromosomes (two signals, each on a 22q11.2 region), which did not present any structural rearrangement (Figure 1E). In patient D, the loss of one red signal was detected in metaphase (Figure 1F), confirming a deletion in the 22q11.2 region of one of her chromosomes.

**Figure 2:**
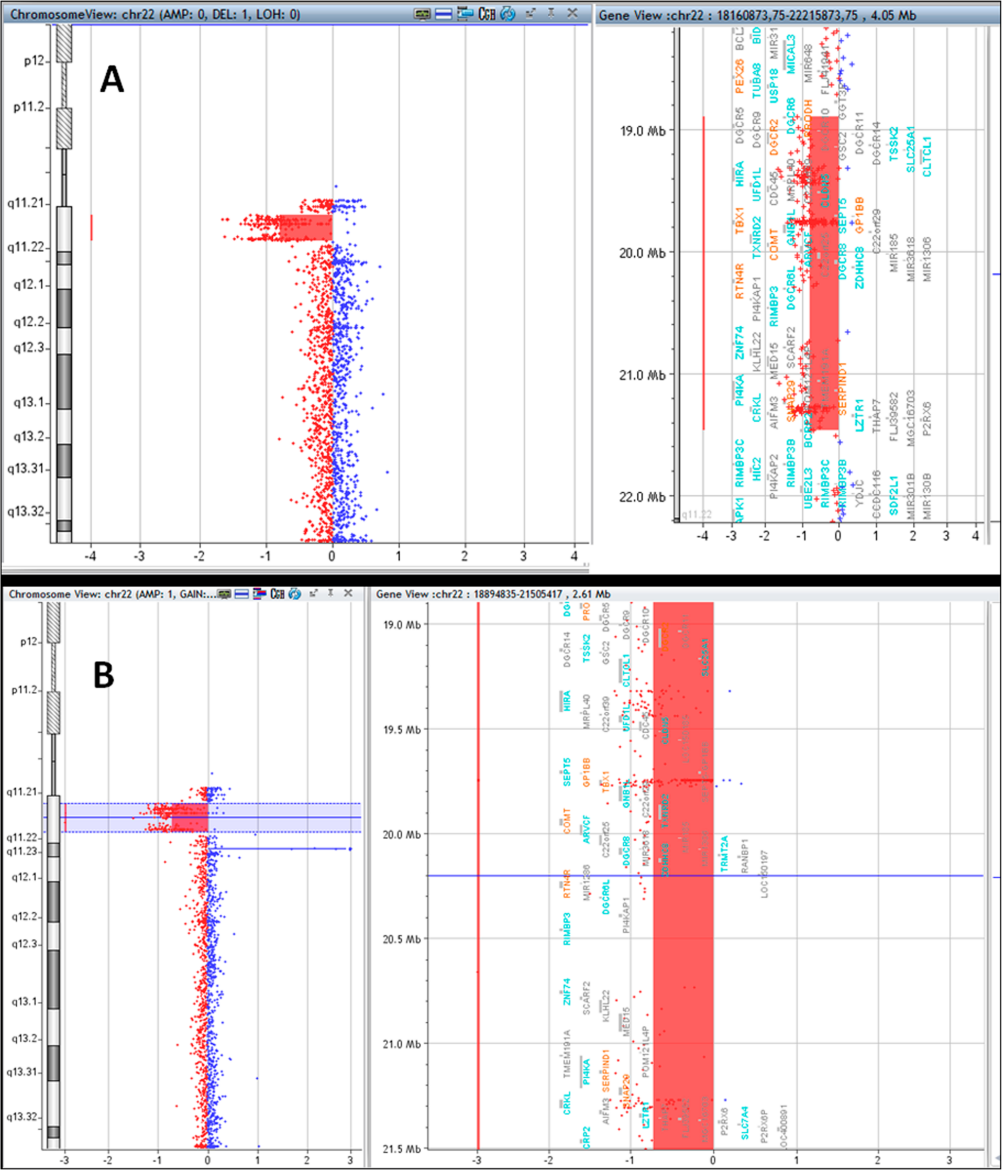
**Graphics of array-CGH assays representing 2.5 Mb deletion within the 22q11.2 region of patient A (A) and patient D (B).**

We additionally performed array-CGH for the determination of the exact length of CNVs in the four patients, as well as in patient B’s father. This technique also allows the identification of the genes involved in the CNV region, using the UCSC Genome Browser. As observed in Figures 2A and 2B, the array-CGH assay detected the 22q11.21 deletion in patients A and D, respectively. The deletions include nucleotide positions 18,894,835 and 21,464,119 for patient A, and positions 18,894,835 and 21,505,417 for patient D, both with an extension of approximately 2.5 Mb. Although having different breakpoints, the deletion in both patients involved the same critical region of the 22q11.2 deletion syndrome (OMIM: #188400; #192430). This region includes 68 genes (UCSC Genome Browser), 39 included in the OMIM database, and 10 in the OMIM Morbid Map (OMIM: _*_606810-*PRODH*, _*_190315-*SLC25A1*, _*_138720-*GP1BB*, _*_602054-*TBX1*, _*_116790-*COMT*, _*_605566-*RTN4R*, _*_613619-*SCARF2*, _*_142360-*HCF2*, _*_604202-*SNAP29*, and _*_600574-*LZTR1*). Despite patient A presented some atypical clinical features for to the 22q11.2 deletion syndrome, such as the hyperactivity, epilepsy, and scoliosis, we did not detect other pathogenic CNVs within his genome.

**Figure 3:**
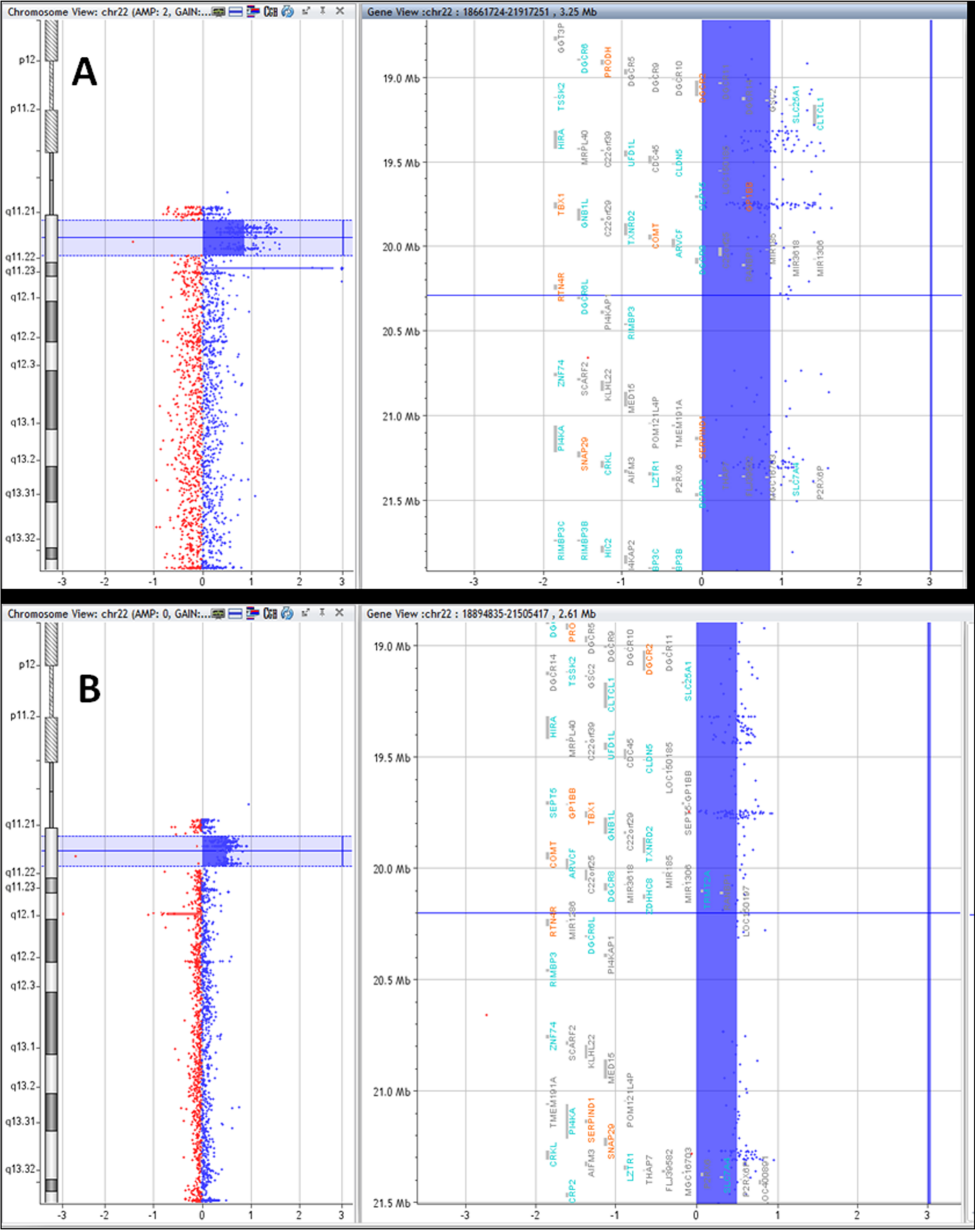
**Representative array-CGH profiles for (A) 22q11.2 triplication of 3 Mb in patient B, and for (B) 22q11.2 duplication of 2.5 Mb in patient B’s father.**

In patient B, we observed the 22q11.2 triplication (Figure 3A), which has an extension of approximately 3 Mb (nucleotide positions 18,661,724 to 21,917,251). Comparing with the genes mapped in 2.5 Mb 22q11.2 deletion, the triplicated region has more eight genes in the UCSC Genome Browser (*i.e.*, 76), more four genes included in the OMIM database (*i.e.*, 43), but the same 10 genes found in the OMIM Morbid Map. The patient B’s father presented a duplication with an extension of approximately 2.5 Mb (Figure 3B; nucleotide positions 18,894,835 to 21,505,417), which is the critical region of the corresponding microduplication syndrome (OMIM: #608363). This duplicated region carries the same genes mapped in the 22q11.2 deletion, namely: 68 in the UCSC Genome Browser, 39 in the OMIM database, and 10 in the OMIM Morbid Map.

**Figure 4:**
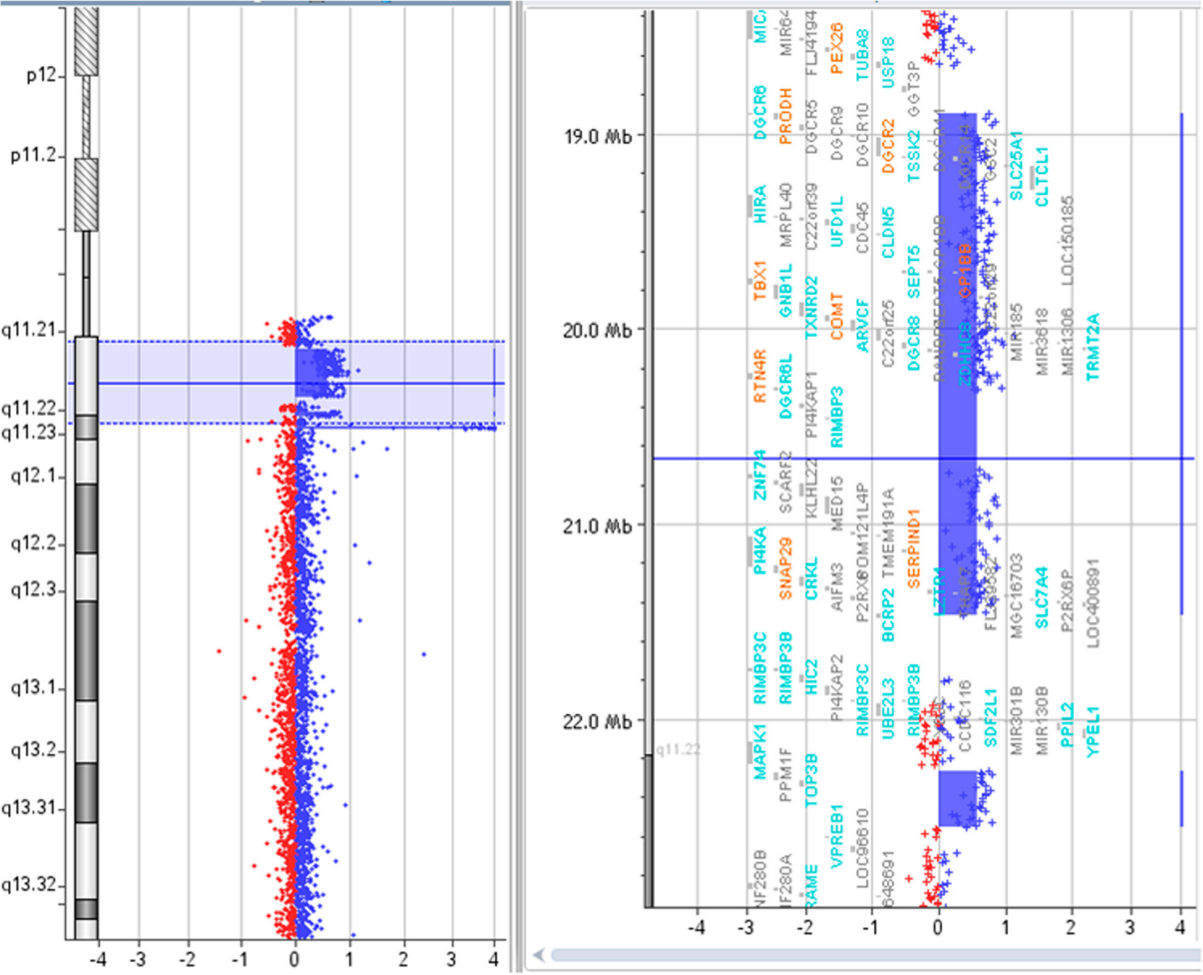
**Analysis of 22q11.2 duplication of 2.5 Mb in patient C.**

Regarding patient C, the array-CGH confirmed a 2.5 Mb duplication of the 22q11.2 region (Figure 4), which was also detected in patient B’s father (Figure 3B). Moreover, we found one duplication in the long arm of chromosome 22, in an extension of approximately 288 Kb (between nucleotide positions 22,261,450 to 22,549,717), which include two genes, *PPMF1* and *TOP3B*, being the last one previously described in the OMIM database (OMIM: _*_603582-*TOP3B*). The downstream extension of the critical region, *i.e. TOP3B* duplication, had been observed by MLPA assay.

## Discussion

Recent reports have shown that CNVs in the 22q11.2 region are detected in a significant proportion of patients with CHD [1,2]. In the present study, we identified new molecular features in CHD patients from the São Miguel Island, Azores. Indeed, out of the 87 patients born alive between 1983 and 2005, four displayed genomic rearrangements in the chromosome 22q11, namely deletions (two patients), duplication (one patient), and triplication (one patient). These results made possible the characterization of the aetiology in these four CHD patients, *i.e.*, 4.6% of our cohort, as well as the association of CNVs with individual clinical features.

The 22q11.2 deletion syndrome has been reported as the most common deletion syndrome [4,9]. With the use of MLPA, molecular cytogenetic and cytogenomic methods, we observed that two unrelated patients (A and D) had this syndrome, both carrying a 2.5 Mb deletion of the 22q11.2 region. The deletion was acquired *de novo*, since their parents showed a normal pattern by MLPA. This observation is in accordance with the literature, where most cases of this syndrome occur after *de novo* deletions [17,18]. Regarding the molecular mechanisms underlying the emergence of both 22q11.2 deletions reported here, we hypothesize to be inter-chromosomal (inter-chromatid) NAHR events in the regions containing LCR sequences, as suggested in the literature [4,5,19]. About 90% of 22q11.2 deletions usually involve a 3 Mb region − the critical region of the 22q11.2 deletion syndrome −, from LCR22-A to LCR22-D [1,2,5]. The 22q11 deletions observed here (2.5 Mb) were included in this region, where the major disease genes are found.

The array-CGH analysis determined that the 22q11.2 deletion included several genes, such as *TBX1*, *COMT*, and *RTN4R*. The *TBX1* gene encodes a T-box transcription factor, and its haploinsufficiency is largely responsible for the physical malformations associated with 22q11.2 deletion syndrome, such as CHD and dysmorphisms [10,20]. This deletion could explain the clinical features of patients A (such as developmental delay, scoliosis, feet bunions, and urogenital abnormality) and patient D (namely developmental delay and hypocalcaemia). Furthermore, the deletion also includes *COMT* and *RTN4R* genes, whose haploinsufficiency is related with mental and behavioural problems [9,19,21]. In fact, patient A presents epilepsy and hyperactivity, whereas patient D has moderate psychomotor impairment. According to the literature, the heart defects most frequently associated with chromosomal abnormalities are VSD, atrioventricular septal defect, and tetralogy of Fallot (TOF) [11]. Interestingly, our results corroborate this observation, since patients A and D, who are affected with the 22q11.2 deletion syndrome, presented a VSD.

The third patient (patient B) carried a 22q11.2 tetrasomy, with one copy in one chromosome and three copies in the other (1:3). This triplication could be explained by NAHR or by unequal crossing-over mechanism during gametogenesis, mediated by the presence of LCRs in the 22q11.2 region [22]. Nevertheless, other hypotheses for the emergence of a triplication are also possible, namely the replication-based mechanisms that underlie complex rearrangements, such as break-induced replication, fork stalling and template switching and/or microhomology-mediated break-induced replication [23,24]. The evaluation of patient B’s parents revealed a 2.5 Mb duplication in her father, suggesting a paternal inheritance with an extra copy. This mode of inheritance may be supported by results from a short tandem repeats/single nucleotide polymorphisms (STRs/SNPs) approach, the only strategy that determines the parental origin of *de novo* CNVs. Despite the different sizes between the CNVs of patient B (3 Mb) and her father (2.5 Mb), we observed an overlapping of the same 10 disease-causing genes, presently registered in the OMIM Morbid Map, but in different gene dosages: patient B has the expression of four gene copies (1:3), whereas her father has three (1:2). To the best of our knowledge, patient B is the second documented case of a 22q11.2 triplication, since the first one was previously described by Yobb and collaborators [14]. These authors reported that the 22q11.2 triplication caused mild phenotypes, including dysmorphic features, hearing impairment, cognitive deficit, behavioural problems, and left ear pit. In the present study, the patient displayed the typical dysmorphic facial features seen in patients with the 22q11.2 deletion and microduplication syndromes, cognitive deficit, and heart defects (a restrictive interventricular communication and a membranous sub-aortic stenosis). Interestingly, these clinical features are similar to those of 22q11.2 deletion syndrome patients but, because include heart defects, they are not considered mild. Taking this into consideration, we propose that 22q11.2 triplication is a variation of 22q11.2 microduplication syndrome, with an aggravated phenotype due to the major dosage of implicated genes.

The last patient (patient C) presented the 22q11.2 microduplication syndrome, carrying a *de novo* 2.5 Mb duplication of this critical region, which could probably be generated during gametogenesis by NAHR [4,19,25] or by unequal crossing-over [8]. To determine the parental origin of this CNV, the same STRs/SNPs strategy suggested for patient B would be useful. This syndrome has also been characterized as a different clinical entity with features overlapping 22q11.2 deletion syndrome [6]. Duplications are associated with high phenotypic variability, including intellectual disability, cognitive deficit, hypotonia, developmental delay, and cardiac heart defects (including VSD, TOF, hypoplastic left heart syndrome, and interrupted aortic arch), as well as normal or near normal phenotype [6,8]. Although carrying the same duplication, patient C and the father of patient B presented different phenotypes. Patient C exhibited ASD, mild developmental delay, and learning difficulties, whereas patient B’s father only has mild intellectual disability with learning difficulties (without CHD). These highly variable cardiac phenotypes and discrepancies could be explained by altered *TBX1* expression together with other additional genetic, epigenetic, and/or non-genetic factors required for full expressivity [8]. Thus, the great variability of the 22q11.2 microduplication syndrome’s severity is the major difficulty in the establishment of genotype-phenotype correlation in the 22q11.2 region. Moreover, in patient C, the short duplication of 288 Kb, which included the *TOP3B* gene, was inherited from her healthy mother and has been described as benign because it was detected in control individuals [26]. However, we cannot exclude the possibility of a combination of the 2.5 Mb 22q11.2 region and *TOP3B* gene duplications for the manifestation of such kind of phenotypes in patient C.

According to Sørensen and collaborators [2], the MLPA technique could be used within paediatric cardiology as a first tier screen to detect clinically relevant CNVs and to identify syndromic patients at an early stage. In the present study, all patients were examined with more than one method and, in each case, the results concurred. The advantage of the MLPA assay compared to FISH and array-CGH is that it is relatively simple to use in clinical laboratories of small or medium-scale dimension, and the reagent cost per assay is cheaper than the other two methods [2,5,27,28]. However, MLPA Kit P250 is not thorough enough for the accurate determination of the sizes of 22q11.2 CNVs and only allows the detection of unbalanced chromosomal rearrangements. As a consequence, the complementary use of array-CGH is advised, since this method is able to perform the fine mapping of the 22q11.2 region. FISH could be required for the screening of balanced rearrangements, essentially in terms of accurate familial genetic counselling [29], and for *in situ* validation of results. Moreover, the aetiology of CHD patients without CNVs needs other molecular analyses, such as the large-scale mutation screening by exome and cDNA sequencing or a strategy based on the analysis of SNPs in candidate genes. We are planning to use some of these techniques to further study the remaining 83 CHD patients without 22q11.2 CNVs.

## Conclusions

In summary, this report is the first study investigating the molecular basis of CHD among patients from the São Miguel Island. Based on a molecular approach combining MLPA, FISH, and array-CGH techniques, we identified CNVs in the 22q11.2 region of four patients, 4.6% of our cohort: two *de novo* deletions (patients A and D), one *de novo* duplication (patient C), and one triplication paternally inherited with an extra copy (patient B). To the best of our knowledge, the latter patient is the second documented case of a 22q11.2 triplication. The data presented here also suggest that triplications of chromosome 22q11.2, although they share some characteristic features with deletion and microduplication syndromes, represent a more severe variant, which are genomically distinct from these well-known syndromes. Finally, the detected CNVs emphasize the relevance of biomedical research, since it can help paediatricians and other professionals to better assess health care needs.

## Methods

### Ethical statement

This work was approved by the Health Ethics Committee from the Hospital of Divino Espírito Santo of Ponta Delgada, EPE (HDES). Written informed consent for patients’ inclusion in the study was obtained from their parents or from the patient himself/herself when he/she had at least 18 year-old. All the parents included in this work also authorized their own participation. Additionally, individuals with CNVs and their parents also gave informed consent to publish their medical and/or genetic information.

### CHD patient screening strategy

The present study was based on the Azorean Registry of CHD, established in 1992, at the HDES, the main hospital in the Azores Islands. The study population consisted of 87 children with heart disease, born alive between 1983 and 2005 in São Miguel Island. Most of the patients were previously included in an epidemiological study of our research group [16].

### MLPA

Genomic DNA from all patients and parents was extracted from peripheral blood lymphocytes using Puregene Blood Kit (Gentra Systems, Minneapolis, USA) or Citogene Blood Kit (Citomed, Lisbon, Portugal), following the manufacturer’s instructions. DNA concentration and purity were evaluated using Beckman DU 530 (Beckman Coulter, Brea, USA) or NanoDrop1000 spectrophotometer (Thermo Scientific, Waltham, USA). The study of the 22q11.2 region was performed by MLPA, using the P250-B1 Kit (MRC-Holland, Amsterdam, The Netherlands) and according to the standard protocol supplied by the manufacturer [30]. A mix of 1 μl PCR product, 9.2 μl Hi-Di formamide and 0.2 μl GeneScan 500 LIZ Size Standard was analyzed by capillary electrophoresis in an ABI 3130 Genetic Analyzer (Applied Biosystems, Foster City, USA). MLPA data were collected using GeneMapper software (Applied Biosystems, Foster City, USA), and subsequently analyzed against up to four control samples with the MRC Coffalyser v7 software.

### FISH

FISH analysis of chromosome 22q11.2 was performed in metaphases and/or interphases, obtained from the synchronous culture of lymphocytes, using the commercially available probe N25 (locus D22S75; Cytocell, Cambridge, UK). Slide preparation, denaturation, and hybridization were carried out according to the manufacturer’s protocols.

### Array-CGH

Genomic DNA extraction from peripheral blood lymphocytes was carried out with the Jetquick Blood and Cell Culture DNA Midi Spin Kit (Genomed, Löhne, Germany), according to the manufacturer’s instructions. DNA concentration and purity were evaluated using a NanoDrop1000 spectrophotometer. Array-CGH was performed for fine mapping of the 22q11.2 region, using the Agilent SurePrint G3 Human Genome Microarray 4X180K (Agilent Technologies, Santa Clara, USA). Labeling and hybridization were carried out with sex-matched reference DNA by using an Agilent Genomic DNA Enzymatic Labeling Kit (Agilent Technologies, Santa Clara, USA), according to the manufacturer’s instructions. Array slides images were acquired on an Agilent scanner and the data were processed with Feature Extraction software (v10.7). Results were analyzed with Agilent Genomic Workbench (v6.5) and Agilent Cytogenomics (v2.7.7.0), according to Human Genome build 19, and interpreted by databases consultation. Regions of copy number change were interpreted with the aid of the UCSC Genome Browser [31].

## Abbreviations

CHD: Congenital heart disease
CNVs: Copy number variants
NAHR: Non-allelic homologous recombination
LCRs: Low-copy repeats
MLPA: Multiplex ligation-dependent probe amplification
FISH: Fluorescent *in situ* hybridization
array-CGH: Array comparative genomic hybridization
VSD: Ventricular septal defect
ASD: Atrial septal defect
TOF: Tetralogy of Fallot
STRs/SNPs: Short tandem repeats/single nucleotide polymorphisms
HDES: Hospital of Divino Espírito Santo of Ponta Delgada, EPE
